# Posterior Occipitocervical Fixation and Intrathecal Baclofen Therapy for the Treatment of Basilar Invagination with Klippel–Feil Syndrome: A Case Report

**DOI:** 10.3390/medicina60050755

**Published:** 2024-05-01

**Authors:** Hitoshi Tonomura, Masateru Nagae, Hidenobu Ishibashi, Kunihiko Hosoi, Takumi Ikeda, Yasuo Mikami, Kenji Takahashi

**Affiliations:** 1 Department of Orthopaedics, Graduate School of Medical Science, Kyoto Prefectural University of Medicine, Kawaramachi-Hirokoji, Kamigyo-ku, Kyoto 602-8566, Japan; 2 Department of Rehabilitation Medicine, Japanese Red Cross Kyoto Daiichi Hospital, 15-749 Honmachi, Higashiyama-ku, Kyoto 605-0981, Japan; 3 Department of Rehabilitation Medicine, Graduate School of Medical Science, Kyoto Prefectural University of Medicine, Kawaramachi-Hirokoji, Kamigyo-ku, Kyoto 602-8566, Japan

**Keywords:** Klippel–Feil syndrome, basilar invagination, occipitocervical fixation, intrathecal baclofen therapy, spastic tetraplegia, opisthotonus

## Abstract

Klippel–Feil syndrome (KFS) is characterized by the congenital fusion of the cervical vertebrae and is sometimes accompanied by anomalies in the craniocervical junction. In basilar invagination (BI), which is a dislocation of the dens in an upper direction, compression of the brainstem and cervical cord results in neurological defects and surgery is required. A 16-year-old boy diagnosed with KFS and severe BI presented with spastic tetraplegia, opisthotonus and dyspnea. CT scans showed basilar impression, occipitalization of C1 and fusion of C2/C3. MRI showed ventral compression of the medullocervical junction. Posterior occipitocervical reduction and fusion along with decompression were performed. Paralysis gradually improved postoperatively over 3 weeks. However, severe spasticity and opisthotonus persisted and intrathecal baclofen (ITB) therapy was initiated. Following this, opisthotonus disappeared and spasticity of the extremities improved. Rehabilitation therapy continued by controlling the dose of ITB. Five years after the surgery, self-propelled wheelchair driving was achieved and activities of daily life improved. The treatment strategy for patients with BI and congenital anomalies remains controversial. Posterior reduction and internal fixation using instrumentation were effective techniques in this case. Spasticity control achieved through a combination of surgery and ITB treatment enabled the amelioration of therapeutic efficacy of rehabilitation and the improvement of ADL.

## 1. Introduction

Klippel–Feil syndrome (KFS) is characterized by the congenital fusion of the cervical vertebrae caused by failure of segmentation [1]. The syndrome sometimes accompanies skeletal anomalies in the craniocervical junction, and it has been reported that some patients develop basilar invagination (BI) with occipitalization of the atlas and spontaneous fusion of C2/3 [2]. In these patients, the odontoid process can migrate upward causing compression of the brainstem and the spinal cord. As a result, various progressive neurological defects develop and operative treatment is necessary. However, for cases of malformations of craniovertebral junction that are severely symptomatic with BI, no standard treatment strategy, including postoperative care, has been established. We experienced the case of a 16-year-old boy with spastic tetraplegia due to KFS. Posterior occipitocervical fixation and intrathecal baclofen (ITB) therapy resulted in a satisfactory outcome in this patient.

## 2. Case Presentation

The patient was a 16-year-old boy. The chief complaint was quadriplegia and dyspnea. In the perinatal period, he was diagnosed with situs inversus and patent ductus arteriosus. Spastic gait appeared when he was a child, and it gradually worsened. Achilles tendon lengthening was performed at the age of 12 and, as a consequence, his gait improved. At the age of 15, quadriplegia and gait disturbances appeared. Dyspnea developed gradually 1 month prior to presentation and his ability to walk declined.

On physical examination, he was alert. Marked head and neck retraction and type II respiratory insufficiency were present. He had hypesthesia on both upper limbs and feet. Muscle weakness at the level of 1–3 measured by manual muscle testing (MMT) was also noted in bilateral arms and legs. There was marked hyperreflexia and severe spasticity. The modified Ashworth scale (MAS) was used to grade spasticity during a physical examination [3]. His average MAS score was 3.6 in the preoperative status. He exhibited a tendency to go into a dystonic position at the slightest touch. This dystonic position was referred to as opisthotonus [4]. He was unable to stand or walk and, as a result, remained in the seated position. His neurological status was evaluated using the Japanese Orthopedic Association scoring system for cervical myelopathy (JOA score). Activities of daily living (ADL) were also evaluated using the motor subscale of the Functional Independence Measure (motor-FIM). His preoperative JOA score was 3.5/17 and his motor-FIM was 15/91.

**Figure 1:**
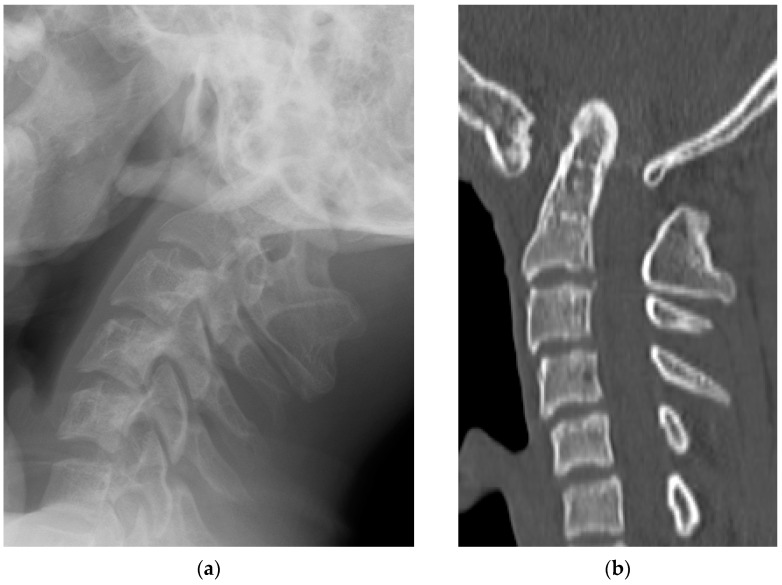
Preoperative radiographic images: (**a**) lateral X-ray view; (**b**) sagittal view of a computed tomography scan.

**Figure 2:**
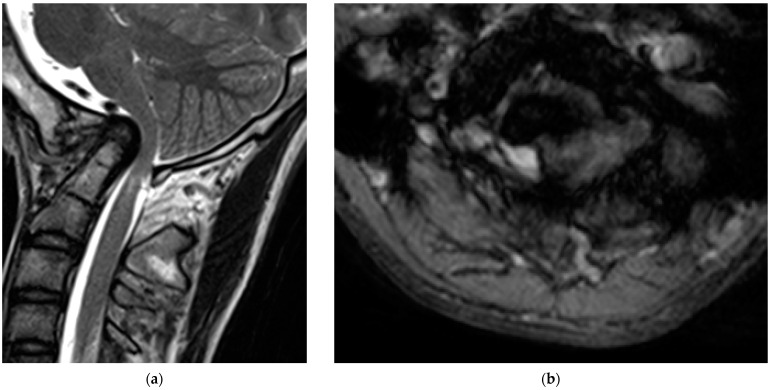
Preoperative magnetic resonance images: (**a**) sagittal; (**b**) axial view of the T2-weighted images.

Radiological examinations demonstrated the abnormalities of the craniocervical junction and fusion of C2/3. Scoliosis in the cervicothoracic region was also confirmed. Computer tomography (CT) scans revealed the assimilation of the occiput with the atlas and posterior deviation of the odontoid process, which was invaginated into the foramen magnum (Figure 1). Magnetic resonance imaging (MRI) demonstrated severe ventral compression of the medulla oblongata and the spinal cord (Figure 2).

**Figure 3:**
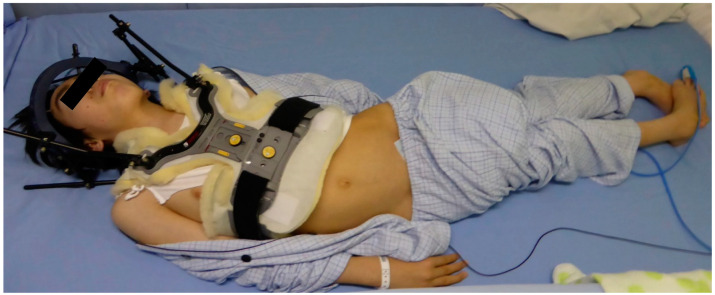
Preoperative patient’s appearance. After hospitalization, a halo vest was fitted and skull traction was performed. Spastic tetraplegia and opisthotonus were present.

After hospitalization, a halo ring (ReSolve halo system; Össur Americas, Inc., Irvine, CA, USA) was fitted and skull traction was attempted with the patient in the conscious state to confirm the potential for reduction. Traction was performed every 4 h for 3 weeks. Following this, respiratory insufficiency resolved and partial vertical reduction was achieved radiologically. However, improvement of symptoms such as muscle weakness, spastic tetraplegia and opisthotonus persisted along with an apparent BI despite the attempts at skull traction (Figure 3). Posterior occipitocervical fixation was then decided on as the next step in treatment.

**Figure 4:**
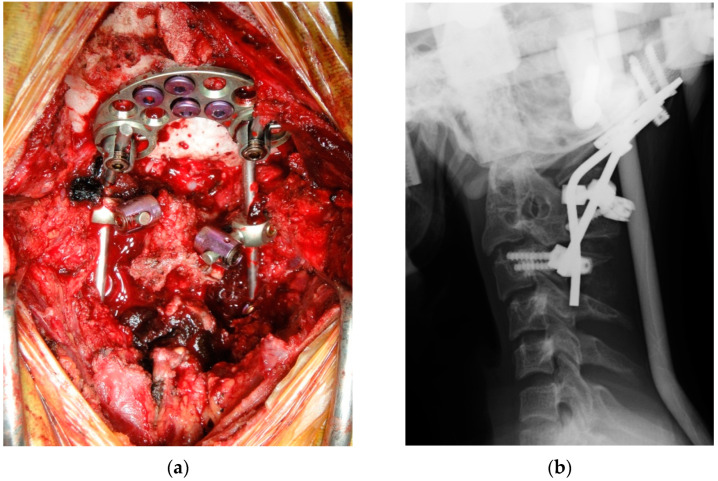
(**a**) Intraoperative findings of posterior occipitocervical fixation; (**b**) postoperative lateral X-ray.

The surgery was performed under general anesthesia in a prone position. Electrodes for muscle evoked potentials (MEPs) following transcranial electrical stimulation (NVM5 nerve monitoring system; NuVasive, Inc., San Diego, CA, USA) were placed on the skull and extremities. A posterior midline longitudinal incision was made from the external occipital protuberance to the spinous process of C6. The occipital bone and cervical lamina were exposed bilaterally. The bone union between the posterior occipital and atlas was exposed and lamina from C2/3 was also fused. There was obvious left–right asymmetry in the posterior occipital shape to the upper cervical lesion. Screws were inserted into the bilateral C2/3 laminar and C4 pedicles under CT-guided navigation (Stealth station S7; Medtronic, Inc., Minneapolis, MN, USA). The occipital plate was fixed using bicortical screws into the thick central part of occiput, and prebent rods were connected temporarily between the occipital plate and cervical screw heads (Vertex; Medtronic, Minneapolis, MN, USA). Decompression of the foramen magnum and C1 laminectomy were performed. A reduction maneuver was performed under fluoroscopy using the following two steps: (1) anterior and (2) inferior reduction of the C2-odontoid process relative to the foramen magnum. A temporary rod holder was placed caudal to the occipital plate. A distractor instrument between the occipital plate and rod holder was used to facilitate anterior reduction. Using the distraction between the rod holder and the C2/3 laminar screw, inferior reduction was achieved (Figure 4) [4]. After decortication, the bone graft harvested from the iliac bone was transplanted and fixed in rods with high-density polyethylene tape (Nesplon; Alfresa, Inc., Osaka, Japan) in order to keep the graft immobilized. No abnormal waveforms were observed on the intraoperative MEP monitoring system.

**Figure 5:**
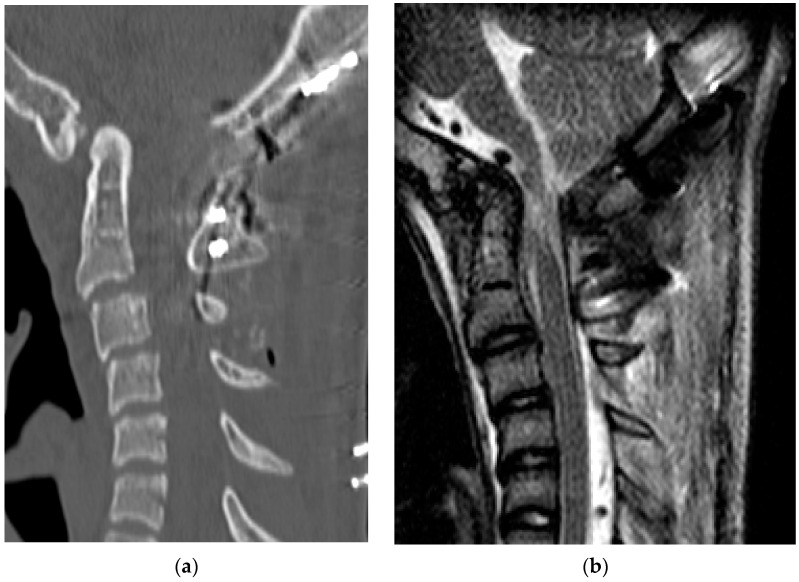
Preoperative images: (**a**) sagittal view of a computed tomography scan; (**b**) sagittal view of the T2-weighted magnetic resonance images.

The halo vest was removed and a neck brace was fixed after the surgery. The patient was kept in an intensive care unit to confirm the absence of airway obstruction and dysphagia. The day after the surgery, the patient was moved to the general ward where ambulation commenced immediately. Radiographs confirmed the reduction of the odontoid process. Postoperative MRI results showed good decompression of the medulla oblongata and the spinal cord (Figure 5). Muscle weakness and numbness of the upper limbs gradually improved postoperatively over 3 weeks. However, severe spasticity and opisthotonus persisted and the average MAS score was 3.4. These symptoms were resistant to oral antispasmodic medications and, as such, it was difficult to perform rehabilitation therapy efficiently. Therefore, we decided to start ITB therapy for 4 weeks after the surgery.

An ITB pump (Synchromed II; Medtronic, Inc., Minneapolis, MN, USA) implantation was performed in this patient who showed a definitive positive effect to the baclofen screening trial and also experienced no adverse events. To further control the spasticity of his upper extremity, an intrathecal catheter tip was applied up to the level of T5. A continuous ITB infusion was started at 50 μg/day. The dose of baclofen was adjusted appropriately with the aim of controlling the level of spasticity on the MAS score so that the score was less than 2.0 and rehabilitation continued simultaneously. One year after the surgery, muscle weakness in the bilateral arms and legs had improved at the level of 3–4 measured by MMT. The dose of baclofen in ITB therapy was gradually increased to 70 μg/day and the average MAS decreased to 1.5. As a result, he could move using a powered wheelchair and have a meal using a spoon unassisted. Radiographs confirmed a union of the bone graft from the occiput to C3. Self-propelled wheelchair driving was achieved 5 years after the surgery. ITB therapy was continued with regular 3–4 month visits to evaluate spasticity and adjust baclofen dosage. He is now 7 years postoperative and is able to live independently in a wheelchair with the JOA score of 5/17 and the motor-FIM of 55/126. The average MAS score is 1.3 and the final baclofen dose is 77 μg/day.

## 3. Discussion

KFS is a congenital union of the cervical vertebrae and its clinical features include the triad of a low posterior hairline and a short neck with limited movement. This syndrome frequently accompanies skeletal anomalies such as scoliosis, Sprengel deformity, hypoplasia of the upper limbs, facial anomalies, basicranial malformation, atlas assimilation and dens malformation. The syndrome also presents with associated disorders including deafness and cardiovascular and genitourinary abnormalities. It has been reported that fewer than half of the patients with KFS exhibit the classical triad. Our case did not present with the classical triad; however, his syndrome was associated with cardiovascular abnormality. In KFS, BI with occipitalization of the atlas and spontaneous fusion of C2/3 is a rare finding [2]. In this case, the patient presented with congenital BI and, as a result, he experienced mild symptoms that were left undiagnosed in childhood. However, his neurological symptoms exacerbated rapidly after the age of 15. It has been reported that the neurological symptoms due to BI often occur at an age between 10 and 20 years. The reason behind this can be attributed to the instability of the occipitocervical junction that causes ligamentous laxity with advancing age. Recent advances in our understanding of basilar invagination associated with craniocervical malformations, including KFS, have shown that mechanical instability is the cause [5]. Further evidence is needed to elucidate the pathology and establish standard treatment guidelines.

Severe neurological defects due to BI should be surgically treated. In surgery, there is a controversy regarding whether an anterior or posterior approach should be used [6,7,8,9]. Management should account for both compression of the brain stem and the spinal cord as well as for craniocervical instability. Anterior decompression by odontoidectomy is achieved using the transoral approach or mandibular split. This procedure enables the spinal cord to displace anteriorly, and has been recommended for patients with severe ventral compression of the medulla oblongata or the spinal cord. However, the anterior surgery is technically demanding and associated with complications such as cerebrospinal fluid leak, infection, respiratory tract obstruction and vertebral arterial injury [9]. An entirely posterior approach to surgery for both reduction and fixation have been reported [6,10]. Spinal cord decompression could be insufficient if the reduction is not successful. Therefore, a detailed evaluation prior to surgery for craniocervical instability is essential for good clinical results. Goel et al. proposed the algorithm for management of BI cases, and the presence of craniocervical instability characterized by vertical mobility and reducibility were the key to determining the surgical procedure [10]. This case presented with craniocervical instability and the BI was partially reducible in the vertical plane. According to Goel’s algorithm, we chose the posterior instrumented reduction and fixation. An intraoperative distraction maneuver was used to achieve the reduction of the BI [6].

In this patient, the injury of the medulla oblongata and the cervical cord due to BI did not only cause tetraplegia but also respiratory insufficiency. Spasticity worsened and, eventually, opisthotonus appeared. Halo vest fixation and skull traction prevented the progression of neuroparalysis and the amelioration of muscle weakness was observed after posterior instrumented surgery. However, muscle spasticity and opisthotonus persisted, which resulted in a challenge for the nursing team and it severely limited the benefits of rehabilitation. Opisthotonus is a relatively rare and severe neurological symptom of spasticity or dystonia that most often results from extended brain ischemia, for example after near-drowning, an asphyxia or a cardiac arrest [4]. In rare cases, opisthotonus occurs due to craniocerebral trauma. These patients show a combination of spasticity and a tendency to go into a dystonic position at the slightest touch. This dystonic position is often referred to as opisthotonus (Greek: opistho = behind and tonus = tone). As a result of this dystonic position and because extreme muscle contractions are easily triggered by touch, nursing and rehabilitation are severely limited. This also leads to secondary joint deformation. Classical pharmacological treatments for opisthotonus include oral administration of benzodiazepines or muscle relaxants. However, successful control of opisthotonus is difficult using these medicines alone. In this patient, oral administration failed to achieve an effective alleviation of muscle spasticity.

ITB therapy has become a well-known treatment for severe spasticity of the cerebral and spinal origin. Several studies reported that severely spastic patients who received ITB showed a marked improvement in their ability to perform ADL and their quality of life [11,12]. Baclofen is administered to the intrathecal space by means of an implantable pump and catheter system, allowing programming of very precise doses. Ceulemans et al. reported the results of 11 young patients with clearly defined opisthotonus and spastic quadriplegia who were treated with ITB therapy. They concluded that ITB therapy is safe and effective for all patients [4]. As soon as ITB administration commenced, abnormal muscle tone decreased remarkably and opisthotonus resolved in our patient. Consequently, an amelioration in patient comfort was achieved, nursing duties could be performed with ease and rehabilitation was successful in improving ADL. Although the timing of ITB initiation is arguable [13], ITB treatment should be considered in young patients with severe spasticity such as opisthotonus.

## 4. Conclusions

To conclude, based on our experiences from this case, we would suggest that cases of KFS that present with severe BI should be carefully evaluated prior to deciding on the course of treatment. Our case and experiences were unique because spasticity control was achieved by a combination of surgery and ITB treatment, which enabled the amelioration of therapeutic efficacy of rehabilitation and the improvement in ADL.

## Data Availability

The data sets used in the present study are available from the corresponding author on reasonable request.
